# Accuracy and Reliability of Intraoral 3D Scans for Diagnostic Evaluations in Nursing Home Residents

**DOI:** 10.1111/ger.12817

**Published:** 2025-03-31

**Authors:** Basel Kharbot, Maike Riegel, Falk Schwendicke, Sebastian Paris, Gerd Göstemeyer

**Affiliations:** ^1^ Department of Operative, Preventive and Pediatric Dentistry Charité – Universitätsmedizin Berlin Berlin Germany; ^2^ Department of Conservative Dentistry and Periodontology Ludwig‐Maximilians‐Universität München Munich Germany

**Keywords:** digital dentistry, geriatric dentistry, intraoral scanner, nursing home, oral diagnostic, telemedicine

## Abstract

**Objectives:**

To evaluate the accuracy and reliability of intraoral 3D scans for assessing the oral health of older patients living in nursing homes.

**Materials and Methods:**

One examiner recorded missing teeth, restorations, caries lesions and oral hygiene (Geriatric‐Debris‐Index Simplified [GDI‐S] ≥ 1.9) in nursing home residents using visual‐tactile diagnostics (reference test) and afterwards obtained intraoral scans with an intraoral scanner (TRIOS 4, 3Shape). Two other independent investigators and the clinical examiner assessed only the scans to delineate the same diagnostic outcomes. For these outcomes, we assessed accuracy, sensitivity, specificity and the Area‐under‐the‐Receiver‐Operating‐Characteristics Curve (AUROC). Kappa values (*κ*) were calculated to evaluate inter‐examiner and intra‐examiner reliability after re‐examination by all examiners after a minimum interval of 3 weeks.

**Results:**

Forty‐three partially dentate patients (65–95 years of age) in need of care living in four nursing homes with a total of 486 teeth (mean [SD]: 11 [9] teeth per patient) were examined. Scans were perfectly accurate for detecting missing teeth (AUROC [sensitivity/specificity]: 1 [1/1]) and showed high accuracy for detecting restorations (0.96 [0.93/0.98]), too. Accuracy was lower to detect caries (0.77 [0.58/0.97]) and insufficient for oral hygiene (0.76 [0.54/0.99]). Agreement between examiners was perfect for missing teeth (*κ*: 1), very good for the detection of restorations (0.94), and good for caries or insufficient oral hygiene (0.73 and 0.72, respectively).

**Conclusions:**

Scans were suitable for basic diagnostic evaluations but showed considerable shortcomings in detecting caries and poor oral hygiene. Assessing scans was relatively reliable.

**Clinical Relevance:**

Using scans may allow telemedical assessments of nursing home residents, but users should be aware of the differential accuracy for different diagnostic targets.

## 1 Introduction

Considerable advances in the prevention and management of oral diseases have led to an overall improvement in oral health in many high‐income countries [1]. However, the ageing population with an increasing number of retained teeth presents a significant challenge to dental healthcare systems, which must provide sufficient oral care to maintain good oral health‐related quality of life in older patients [2, 3]. Consequently, frail or functionally dependent older people are found to have poor oral hygiene and a high experience of the major oral diseases, such as caries and periodontitis. Given their cumulative nature, these diseases also tend to exhibit a higher degree of severity and extent than in younger population groups [4, 5, 6, 7].

In addition to oral hygiene, regular access to dental services is essential for maintaining oral health [8]. However, as older people become increasingly dependent and less mobile, their access to dental services declines while their need for treatment remains high [9]. In fact, most functionally dependent older people are not regularly examined and treated by a dentist. Instead, dental care is typically sought and provided only in emergencies and at a basic level [10, 11].

The use of telemedicine approaches may be promising for improving access to dental care for underserved groups, such as vulnerable patients [12]. A recent study investigated the use of smartphone photographs to assess the oral care status of nursing home residents [13, 14, 15]. While photographic documentation can provide insight into the intraoral care status of functionally dependent older people, there is currently no established method for the extensive telemedical examination of functionally dependent older people. Within this context, the use of intraoral scanners might be promising for telemedical screening and diagnostic purposes in functionally dependent older people: The three‐dimensional recording and visualisation of the mouth could improve the accuracy of assessing dental conditions. In the case of dental caries, the ability to image teeth using fluorescence methods could also contribute to a more accurate diagnosis. To date, several studies have investigated the applicability of intraoral scanners for detecting caries lesions in vitro [13, 14, 15]. However, there is a lack of clinical evidence to support the use of intraoral scanners for the clinical assessment of functionally dependent older people. The objective of this proof‐of‐concept study was to evaluate the accuracy and reliability of 3D scans for telemedical examination of the dental status of functionally dependent older people residing in care facilities.

## 2 Materials and Methods

### 2.1 Study Design

This was a proof‐of‐concept cross‐sectional clinical study. The study was conducted as part of an overarching research project ‘TAILOHR’ funded by the German Federal Ministry of Education and Research (Bundesministerium für Bildung and Forschung (BMBF) grant no.: 01GY1802). Ethics approval was given by the ethics review board of the Charité University on 24.07.2019 (EA4/113/19). Reporting of this study is in accordance with the Standards for Reporting Diagnostic accuracy studies (STARD 2015 Checklist) [16].

The clinical investigations were undertaken between 11/2019 and 02/2020 at four care facilities in the rural region of Brandenburg (Germany). Forty‐five functionally dependent older people aged between 65 and 95 years were recruited and gave informed consent to participate (or their authorised relatives). However, 43 of them were included in the study after one withdrew and one examination was discontinued due to insufficient cooperation.

An examiner ‘Eclin’ undertook the intraoral examinations using visual tactile assessment (reference test). All examinations were conducted in the nursing homes in the participants' rooms, primarily with them in a sitting position. For bedridden participants, a lying position was used. Visual‐tactile examinations were carried out without prior dental cleanings, utilising a dental loupe (EyeMag Smart 2.5‐fold magnification, Zeiss, Jena, Germany) and dental instruments including two mirrors (TOPvision No. 5, Hahnenkratt, Königsbach‐Stein, Germany), a probe (Aesculap DA410R, B. Braun SE, Melsungen, Germany) and tweezers (Aesculap DA225R, B. Braun SE, Melsungen, Germany). The examination comprised recording missing teeth and restorations (y/n), assessing caries lesions using ICDAS II criteria and evaluating plaque using the Geriatric Debris Index Simplified (GDI‐S). For the comparison between the clinical assessment and evaluation of the scans, only cavitated caries lesions (W1/W2 and ICDAS 3–6) were considered. The GDI‐S was scored on the buccal surfaces of all teeth within a 4‐point scale (0–3) reflecting the amount of debris on each tooth. Code 0 corresponds to no plaque while codes 1–3 correspond to plaque on up to 1/3 of the tooth surface or intrinsic discoloration regardless of the amount of plaque (1), 2/3 of the tooth surface (2) or plaque exceeding 2/3 of the tooth surface (3), respectively. To reflect the oral hygiene of the participants, the mean GDI‐S score across all teeth present was calculated. A mean score of < 1.9 was considered adequate oral hygiene and a higher score reflected inadequate oral hygiene. Deeply decayed teeth (ICDAS 5/6) and root remains were uniformly recorded as carious and allocated a GDI‐S score of 3 [17].

Scans were obtained following the clinical examinations using an intraoral scanner (TRIOS 4, 3Shape, Copenhagen, Denmark) based on confocal imaging technology according to the manufacturer's recommendations under ambient light (with no direct light source on the patients mouths) with a predefined scan path, starting from the occlusal surfaces of the full arches (from the distal left side of the patient). In the anterior region, pivoting movements were carried out because incisal surfaces contain little surface information. In the upper jaw, the scanner was turned on the buccal surfaces following the opposite direction back and finally turned on the palatal surfaces and palate starting again from the distal left side of the patient. In the mandible, first the lingual and then the buccal surfaces were scanned. Subsequently, the caries diagnostic aid function of the scanner was activated, and a new scan was taken under fluorescent light (415 nm wavelength) using the same scan path. In the corresponding 3D models, initial caries lesions were highlighted in yellow, whereas moderate or extensive caries lesions were highlighted in red. White areas indicated areas with insufficient scan data. Due to limited cooperation or technical challenges, scanning procedures had to be partially reiterated in a few cases where the full region of interest could not be completely depicted.

**FIGURE 1:**
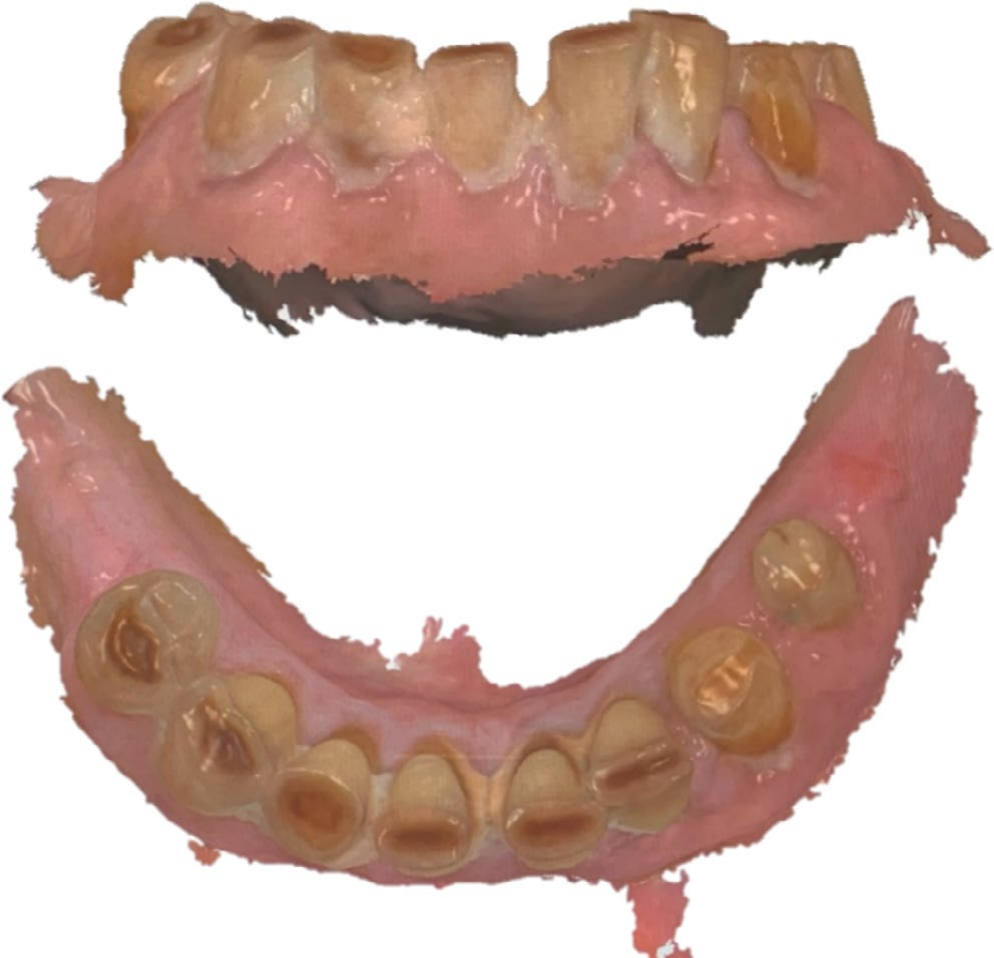
Example depiction of a scanned mandible with clearly visible calculus and/or plaque on all teeth. The possibility of visualising the scans three‐dimensionally facilitates an assessment of the teeth from all perspectives. [Colour figure can be viewed at wileyonlinelibrary.com]

To simulate a telemedicine approach, the scans were subsequently assessed on the scanner screen (Figure 1) by two independent examiners (Esc1 and Esc2) and additionally by the clinical examiner Eclin, with a 10‐month interval between the clinical examinations and assessments. The same parameters were recorded as were done during the clinical examination. The examiners were able to switch between the realistic colour presentation and the scans obtained using the fluorescent diagnostic aid during the assessment. A total of 10 randomly selected 3D scans were re‐examined for the same parameters by all examiners after a minimum interval of 3 weeks in order to calculate intra‐examiner reliability.

### 2.2 Data Analysis

The outcomes were accuracy, sensitivity, specificity and the Area‐under‐the‐Receiver‐Operating‐Characteristics Curve (AUROC) with the clinical investigation of Eclin as reference. Diagnoses were categorised as true positives if they were made both in the clinical and in telemedical examination and as true negatives if made in no examination. Diagnoses were categorised as false positives if made only in the telemedical examination and as false negatives if made only in the clinical examination. Sensitivity marks the proportion of correct positive assignments of the telemedical assessment out of all positive assignments. Specificity marks the proportion of correctly categorised negative assignments of the telemedical assessment out of all negative assignments.

Receiver Operating Characteristic (ROC) curves were generated, and the Area‐under‐the‐Receiver‐Operating‐Characteristics Curve (AUROC) was calculated to visualise and quantify the accuracy of telemedical investigations. Cohen's Kappa (*κ*) was calculated to evaluate inter‐d intra‐examiner reliability.

## 3 Results

The 43 included participants were aged between 65 and 95 (mean [SD]: 81 [8]) years and had a total of 486 teeth (mean [SD]: 11 [9] teeth per patient). An exemplary scan used for telemedical assessment is shown in Figure 1.

**FIGURE 2:**
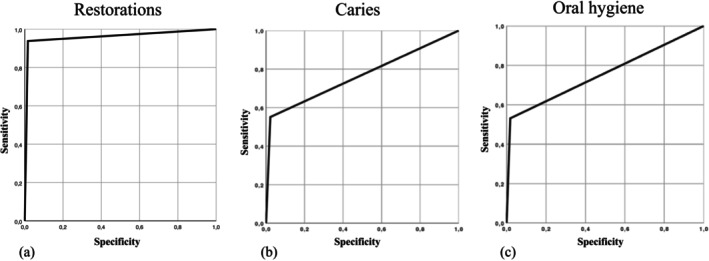
Diagnostic accuracy of the 3D scans visualised by ROC curves for restorations (a), caries (b) and oral hygiene (c). The accuracy of the telemedical caries detection was evaluated against the clinical findings. In the ROC curves, the averaged sensitivity of all examiners (*y*‐axis) is plotted against the specificity (1‐specificity; *x*‐axis). The specificity decreases with increasing sensitivity. The Area Under the Curve (AUC) corresponds to the area below the curve and is an overview measure of accuracy.

**FIGURE 3:**
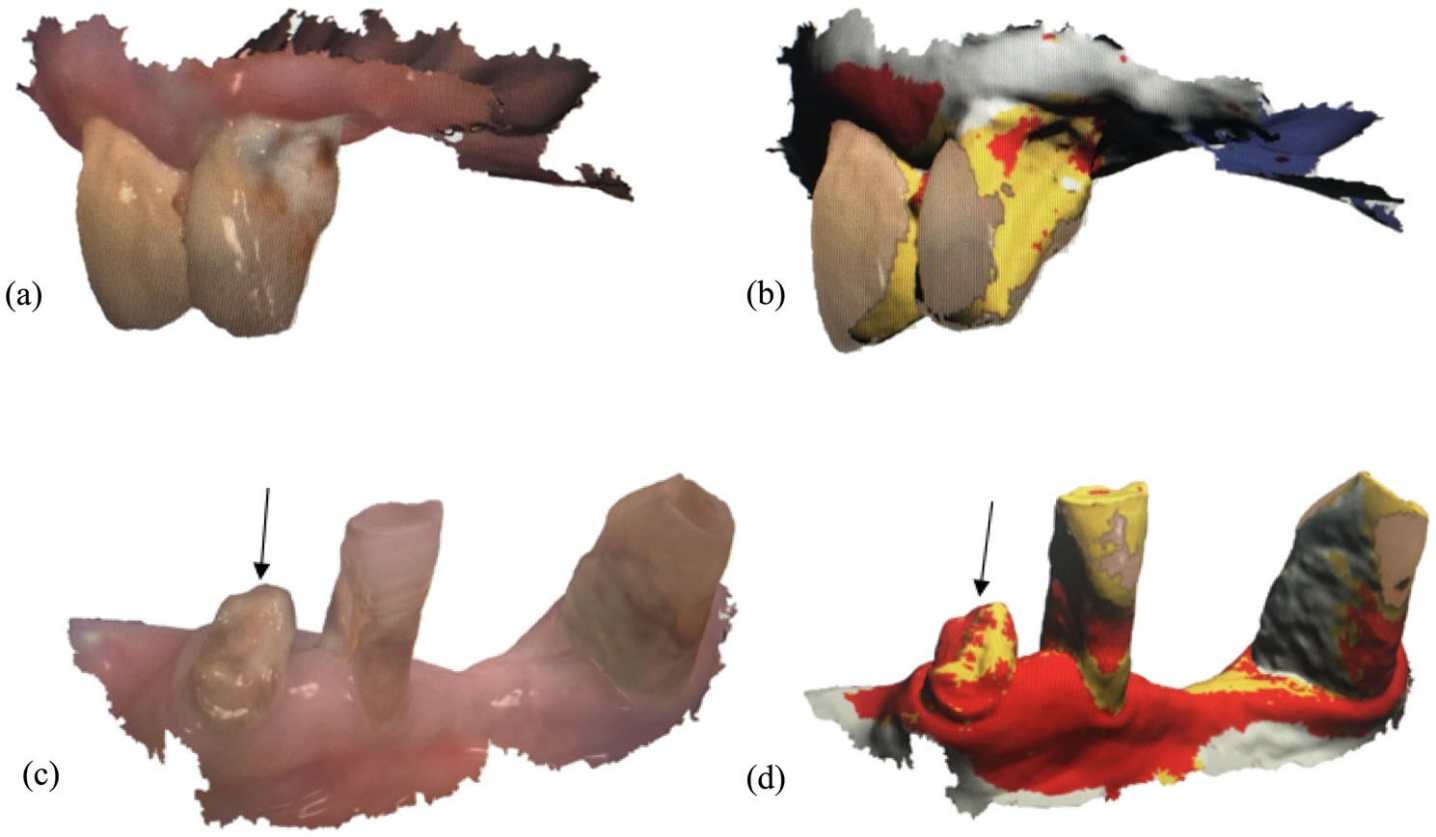
Original depictions (a, c) as well as depiction with additional fluorescence for caries detection in 3D models of an upper and lower jaw (b, d). Original (a) A cavitated caries lesion on the mesial surface of 12 was heavily covered with plaque. Fluorescence (b) Due to the density properties of plaque, the largest part of both teeth were depicted in yellow, leaving only small areas with less plaque on the caries lesion shown in red. Oroginal (c) Due to the presence of plaque, carious areas on the root surfaces of teeth 33 and 42 were difficult to delineate. Fluorescence (d) In region 43, the carious tooth structure was clearly recognisable in yellow‐red (arrow). In region 33, white areas indicated insufficient scan data mesially. Gingival tissue was predominantly shown in red (b). [Colour figure can be viewed at wileyonlinelibrary.com]

The mean accuracy for detecting missing teeth (AUROC [sensitivity/specificity]: 1 [1/1]) and restorations (0.96 [0.93/0.98]) using scans was higher than for detecting caries (0.77 [0.58/0.97]) or insufficient OH (0.76 [0.54/0.99]) (Figure 2), with no relevant differences between examiners detected. Agreement between examiners was perfect for missing teeth (*κ*: 1), very good for detecting restorations (*κ*: 0.94) and good for detecting caries (0.73) or poor oral hygiene (0.72), respectively (Table 1). The tooth‐related assessments of the GDI‐S showed only moderate inter‐ and intra‐rater reliability (0.44/0.60) due to technical limitations of the intraoral scanner (Figure 3). Intra‐rater reliability was very good for detecting restorations (0.95) and good for caries (0.79).

**TABLE 1:** Sensitivity, specificity and Area‐under‐the‐Receiver‐Operating‐Characteristics Curve (AUROC) values of the telemedical assessments of variables.

Variable	Examiner	Sensitivity	Specificity	AUROC	Intra‐rater reliability (*κ*)
Missing teeth	Eclin	1.00	1.00	1.00	1.00
	Esc 1	1.00	1.00	1.00	1.00
	Esc 2	1.00	1.00	1.00	1.00
Restorations	Eclin	0.92	0.96	0.94	0.97
	Esc 1	0.93	0.99	0.96	0.91
	Esc 2	0.94	0.98	0.96	0.96
Caries	Eclin	0.64	0.96	0.80	0.78
	Esc 1	0.51	0.98	0.74	0.79
	Esc 2	0.59	0.98	0.78	0.79
GDI‐S	Eclin	0.56	1.00	0.78	0.61
	Esc 1	0.56	0.96	0.76	0.68
	Esc 2	0.50	1.00	0.75	0.52

## 4 Discussion

Telemedicine holds promise for filling healthcare gaps for underserved patient groups, such as nursing home residents. This proof‐of‐concept clinical study assessed the reliability of scans as a telemedicine approach to evaluate basic diagnostic findings (missing teeth, restorations, caries and plaque) in functionally dependent older people. Due to the inherent limitations of clinical imaging procedures in providing only supragingival information, these diagnostic outcomes were selected to provide the most comprehensive and relevant data regarding the cariologic and functional/prosthetic status of the patients on a basic level. While periodontal findings, oral mucosa findings and other elements are crucial for comprehensive diagnostics, they cannot be captured using an intraoral scanner. The findings of our study indicate a high accuracy as well as reliability for detecting missing teeth and restorations using intraoral scanners. However, this method was less accurate in detecting caries and plaque.

Our study is not without limitations, which we consider as follows. Recruiting study participants proved to be a significant challenge, as the functionally dependent older people were typically in need of care and the study procedures had to be integrated into the daily routine of the care facility. Additionally, the authorised caregivers of the functionally dependent older people, who were often unavailable, were required to provide consent for their participation in the study. These circumstances significantly constrained the study's sample size, which represents an inherent limitation of this study.

Second, it should be noted that no professional dental cleaning was performed prior to the clinical examinations and scanning, allowing for an evaluation of the participants' oral hygiene. The presence of plaque, however, may have influenced the accurate diagnosis of underlying caries lesions. Thus, plaque removal can still be recommended prior to examination in order to ensure a more accurate diagnosis, although this may not always be feasible in routine care. Nevertheless, the scans provide a relatively accurate representation of the true clinical situation of functionally dependent older people, which may be sufficient for a preliminary assessment of treatment need [13].

Moreover, the clinical examinations of the participants in our study were undertaken in the patients' rooms, sitting in a chair, or lying in bed, without the use of a dental unit. Although this represents a realistic setting, the lack of a professional equipment may further increase the risk of inaccurate assessments due to inadequate lighting or suction, which may cause unwanted reflections in the scans due to wet tooth surfaces [18]. Hence, the detection of less accessible areas and metal restorations was more challenging, resulting in partial areas that could not be adequately assessed with the caries diagnostic aid (Figure 3). Furthermore, wet gingiva impeded the collection of coherent data and the orientation of the scanning system. These more relevant technical limitations for screening purposes of patients, as also noted in other studies, resulted in difficulties in the detection of distal teeth adjacent to larger gaps, which partly increased scanning times noticeably [19]. In combination with the limited cooperation of some multimorbid participants, the scanning procedure occasionally proved difficult. Nevertheless, scanning generally seems feasible for trained care staff, although the clinical use of intraoral scanners is currently only permitted by dental professionals in Germany. This requirement had to be fulfilled in order to obtain ethical approval. However, given the noninvasive nature of the intraoral scans and the observed increase in the number of individuals requiring care and treatment, we hope to be able to conduct future studies in which nursing staff will be trained to perform the scans independently.

The clinical examinations that were utilised as a reference for assessing the accuracy of the scans were conducted by a single examiner. This was done to minimise the physical burden on the participants, a strategy that has been employed in similar studies as well [15, 20]. While this approach may introduce a potential risk of bias due to the possibility of incorrect assessments by the clinical examiner, it can be assumed to be low as the evaluation criteria were either dichotomous or well defined (e.g., the presence or absence of restorations and the identification of cavitated caries lesions). Moreover, a calibration of the study's examiners was conducted prior to the investigation to preclude potential bias. Although the rough categorisation into cavitated and noncavitated caries does not necessarily correspond to the current standard, it appears pragmatic for assessing the fundamental treatment requirements of the functionally dependent older people in a first step. Future studies should of course implement more differentiated categorisations to facilitate the development of reliable, individualised treatment strategies based on intraoral scans. Additionally, an auxiliary feature supporting caries diagnostics based on automated fluorescence imaging was employed in this study [14]. However, this did not yield any additional diagnostic value for caries detection in our setting. This finding is consistent with that of a recent study which reported no significant differences in the assessment of 3D models with tooth colour alone or supplemented with fluorescence. While the remote on‐screen assessment of (occlusal) caries lesions demonstrated a comparable performance to that of clinical examinations, the detection of early caries lesions proved considerably more difficult than that of moderate or extensive lesions [21]. Certain intraoral scanners employ the use of near‐infrared light as a diagnostic aid. These devices could have the potential to facilitate a more accurate detection of proximal caries lesions in enamel. However, they are not yet a substitute for radiological diagnostics, which remain the gold standard [22, 23, 24, 25]. Furthermore, it is questionable whether this approach provides any benefit in caries assessment in functionally dependent older people, given that older individuals are less susceptible to proximal caries lesions.

Lastly, the clinical examiner also undertook the on‐screen assessments, which pose a potential risk for (expectation) bias. However, a minimum interval of 10 months between the clinical and telemedical assessments was ensured to minimise the risk of bias. Moreover, the comparison of performances among the examiners allows for exclusion of the possibility that the data may have been distorted due to an insufficient level of calibration among them. Since no systematic differences in examiner performance were identified, it can be concluded that the lower accuracy in caries and oral hygiene detection is attributable to the scans.

The use of intraoral scanners in clinical dental practice has grown considerably in recent years, with an expanding range of applications. These include the capture of optical impressions for the manufacturing or planning of indirect restorations, as well as surgical and orthodontic applications [26]. While scans have inherent limitations in terms of trueness and precision for extended restorations, these may be less of a concern for screening and diagnostic purposes, where greater detail is not required. Instead, limitations for diagnostic purposes arise from inaccurate (frequently brighter) colour reproduction or slightly blurred resolution of the scans when being assessed on screen, as has been documented in other studies [19, 27].

Alternative imaging methods for telemedical screening procedures have already been investigated, including intraoral cameras and conventional smartphones [19]. Although these devices can provide a realistic representation of the intraoral situation, the assessment of findings in a telemedicine context is constrained by the perspective selected at the time of recording and the two‐dimensional representation of the intraoral situation. Inaccuracies in the visualisation of plaque were also observed in a previous telemedicine study using conventional intraoral cameras [28]. This suggests that inaccurate visualisation of oral structures is not limited to the use of scans, but rather affects various telemedicine approaches [28]. However, a recent clinical study reported that current intraoral scanners may be suitable for plaque detection after adaptation of planimetric methods to the colour representation of the intraoral scanners [29]. In the light of the findings from this study and the technological advancements in intraoral scanners and their additive features, it is evident that 3D scans have the potential to emerge as a more valuable method for collecting comprehensive clinical data in telemedical settings when compared with conventional imaging methods. Nevertheless, all methods that rely exclusively on clinical imaging are inherently limited in their ability to provide comprehensive information regarding periodontal, mucosal or temporomandibular joint health, which often require clinical intervention to obtain the necessary information [20].

Perspectively, a critical evaluation of the cost‐effectiveness of a telemedical approach that utilises intraoral scanners is necessary, particularly in the light of the substantial financial investments required for equipment acquisition, the necessity for training, and the allocation of personnel to execute the procedures, and in consideration of the accuracy of the scans and anticipated benefit for the patients. However, technological advancements and declining costs for equipment imply that the necessary investments may become justifiable over time [30]. Furthermore, the majority of studies report a high level of acceptance among patients and dental personnel regarding telemedicine approaches [31, 32, 33, 34].

Overall, the aforementioned limitations of caries diagnostics based on scans (with or without fluorescence‐based assistance) indicate that intraoral scanners for telemedical procedures may be a suitable option for screening and triage purposes or as a supplementary tool to the clinical examination, including diagnostic imaging such as bitewing radiographs [19, 32]. However, it is imperative that clinicians consider the differential accuracy of scans when assessing different targets. It is conceivable that future advancements in scanner technology may enhance the accuracy of the procedure.

## 5 Conclusion

The intraoral scans obtained with the tested device are suitable for diagnostic evaluations, but they are less accurate than clinical examinations for assessing dental caries and oral hygiene. Consequently, intraoral scanners are currently feasible as a screening tool for telemedicine purposes, but not yet as a replacement for clinical examination.

## Author Contributions

**Basel Kharbot:** investigation, formal analysis, writing – original draft, writing – review and editing, visualisation. **Maike Riegel:** investigation, data curation. **Falk Schwendicke:** conceptualisation, methodology, funding acquisition, writing – review and editing. **Sebastian Paris:** funding acquisition, writing – review and editing. **Gerd Göstemeyer:** conceptualisation, methodology, formal analysis, writing – review and editing, supervision. All authors have read and agreed to the published version of the manuscript.

## Ethics Statement

Ethics approval was given by the ethics review board of Charité University of Berlin (EA4/113/19).

## Conflicts of Interest

The authors declare no conflicts of interest.

## Data Availability

The data that support the findings of this study are available from the corresponding author upon reasonable request.

## References

[1] E. Bernabe , W. Marcenes , C. R. Hernandez , et al., “Global, Regional, and National Levels and Trends in Burden of Oral Conditions From 1990 to 2017: A Systematic Analysis for the Global Burden of Disease 2017 Study,” Journal of Dental Research 99, no. 4 (2020): 362–373, 10.1177/0022034520908533. 32122215 PMC7088322

[2] I. Nitschke and W. Micheelis , “Krankheits‐und Versorgungsprävalenzen bei Älteren Senioren mit Pfl egebedarf,” in Fünfte Deutsche Mundgesundheitsstudie (DMS V), ed. A. Jordan and W. Micheelis (Deutscher Zahnärzte Verlag DÄV, 2016), 557–578.

[3] G. Gostemeyer , S. R. Baker , and F. Schwendicke , “Barriers and Facilitators for Provision of Oral Health Care in Dependent Older People: A Systematic Review,” Clinical Oral Investigations 23, no. 3 (2019): 979–993. 30707299 10.1007/s00784-019-02812-4

[4] L. M. De Visschere , L. Grooten , G. Theuniers , and J. N. Vanobbergen , “Oral Hygiene of Elderly People in Long‐Term Care Institutions—A Cross‐Sectional Study,” Gerodontology 23, no. 4 (2006): 195–204. 17105500 10.1111/j.1741-2358.2006.00139.x

[5] R. Jordan and W. Micheelis , Fünfte Deutsche Mundgesundheitsstudie (IDZ, 2015). 10.1186/1472-6831-14-161 PMC4417261 25547464

[6] P. I. Eke , L. Wei , W. S. Borgnakke , et al., “Periodontitis Prevalence in Adults ≥65 Years of Age, in the USA,” Periodontology 2000 72, no. 1 (2016): 76–95. 27501492 10.1111/prd.12145 PMC8223257

[7] R. Lopez , P. C. Smith , G. Gostemeyer , and F. Schwendicke , “Ageing, Dental Caries and Periodontal Diseases,” Journal of Clinical Periodontology 44, no. Suppl 18 (2017): S145–S152. 28266118 10.1111/jcpe.12683

[8] L. A. Crocombe , J. M. Broadbent , W. M. Thomson , D. S. Brennan , and R. Poulton , “Impact of Dental Visiting Trajectory Patterns on Clinical Oral Health and Oral Health‐Related Quality of Life,” Journal of Public Health Dentistry 72, no. 1 (2012): 36–44. 22316176 10.1111/j.1752-7325.2011.00281.x

[9] P. C. Bots‐VantSpijker , J. N. Vanobbergen , J. M. Schols , R. M. Schaub , C. P. Bots , and C. de Baat , “Barriers of Delivering Oral Health Care to Older People Experienced by Dentists: A Systematic Literature Review,” Community Dentistry and Oral Epidemiology 42, no. 2 (2014): 113–121. 24102439 10.1111/cdoe.12068

[10] H. Frenkel , I. Harvey , and R. G. Newcombe , “Oral Health Care Among Nursing Home Residents in Avon,” Gerodontology 17, no. 1 (2000): 33–38. 11203511 10.1111/j.1741-2358.2000.00033.x

[11] M. Albrecht , R. Kupfer , D. R. Reissmann , I. Muhlhauser , and S. Kopke , “Oral Health Educational Interventions for Nursing Home Staff and Residents,” Cochrane Database of Systematic Reviews 9 (2016): CD010535. 27689868 10.1002/14651858.CD010535.pub2 PMC6457754

[12] S. J. Daniel and S. Kumar , “Teledentistry: A Key Component in Access to Care,” Journal of Evidence‐Based Dental Practice 14 (2014): 201–208. 24929605 10.1016/j.jebdp.2014.02.008

[13] S. Michou , R. Benetti Ana , C. Vannahme , G. Hermannsson Pétur , A. Bakhshandeh , and R. Ekstrand Kim , “Development of a Fluorescence‐Based Caries Scoring System for an Intraoral Scanner: An In Vitro Study,” Caries Research 54, no. 4 (2020): 324–335. 33053552 10.1159/000509925

[14] S. Michou , M. S. Lambach , P. Ntovas , et al., “Automated Caries Detection In Vivo Using a 3D Intraoral Scanner,” Scientific Reports 11, no. 1 (2021): 21276. 34711853 10.1038/s41598-021-00259-w PMC8553860

[15] D. Bleiel , T. Rott , I. Scharfenberg , M. J. Wicht , and A. G. Barbe , “Use of Smartphone Photos to Document the Oral Care Status of Nursing Home Residents,” Gerodontology 40, no. 2 (2023): 244–250. 35924660 10.1111/ger.12650

[16] P. M. Bossuyt , J. B. Reitsma , D. E. Bruns , et al., “STARD 2015: An Updated List of Essential Items for Reporting Diagnostic Accuracy Studies,” BMJ 351 (2015): h5527. 26511519 10.1136/bmj.h5527 PMC4623764

[17] M. I. MacEntee , C. C. Wyatt , B. L. Beattie , et al., “Provision of Mouth‐Care in Long‐Term Care Facilities: An Educational Trial,” Community Dentistry and Oral Epidemiology 35, no. 1 (2007): 25–34, 10.1111/j.1600-0528.2007.00318.x. 17244135

[18] H. Kihara , W. Hatakeyama , F. Komine , et al., “Accuracy and Practicality of Intraoral Scanner in Dentistry: A Literature Review,” Journal of Prosthodontic Research 64, no. 2 (2020): 109–113. 31474576 10.1016/j.jpor.2019.07.010

[19] S. Steinmeier , D. Wiedemeier , C. H. F. Hämmerle , and S. Mühlemann , “Accuracy of Remote Diagnoses Using Intraoral Scans Captured in Approximate True Color: A Pilot and Validation Study in Teledentistry,” BMC Oral Health 20, no. 1 (2020): 266. 32977794 10.1186/s12903-020-01255-8 PMC7517740

[20] S. Daly , J. Seong , C. Parkinson , R. Newcombe , N. Claydon , and N. West , “A Proof of Concept Study to Confirm the Suitability of an Intra Oral Scanner to Record Oral Images for the Non‐Invasive Assessment of Gingival Inflammation,” Journal of Dentistry 105 (2021): 103579. 33417977 10.1016/j.jdent.2020.103579

[21] P. Ntovas , S. Michou , A. R. Benetti , et al., “Occlusal Caries Detection on 3D Models Obtained With an Intraoral Scanner. A Validation Study,” Journal of Dentistry 131 (2023): 104457. 36858167 10.1016/j.jdent.2023.104457

[22] I. H. Baltacioglu and K. Orhan , “Comparison of Diagnostic Methods for Early Interproximal Caries Detection With Near‐Infrared Light Transillumination: An In Vivo Study,” BMC Oral Health 17, no. 1 (2017): 130. 29145846 10.1186/s12903-017-0421-2 PMC5689175

[23] A. Marmaneu‐Menero , J. E. Iranzo‐Cortés , T. Almerich‐Torres , J. C. Ortolá‐Síscar , J. M. Montiel‐Company , and J. M. Almerich‐Silla , “Diagnostic Validity of Digital Imaging Fiber‐Optic Transillumination (DIFOTI) and Near‐Infrared Light Transillumination (NILT) for Caries in Dentine,” Journal of Clinical Medicine 9, no. 2 (2020): 420. 32033068 10.3390/jcm9020420 PMC7073697

[24] M. I. G. Ortiz , C. de Melo Alencar , B. L. F. De Paula , M. B. Magno , L. C. Maia , and C. M. Silva , “Accuracy of Near‐Infrared Light Transillumination (NILT) Compared to Bitewing Radiograph for Detection of Interproximal Caries in the Permanent Dentition: A Systematic Review and Meta‐Analysis,” Journal of Dentistry 98 (2020): 103351. 32380136 10.1016/j.jdent.2020.103351

[25] E. Stratigaki , F. N. Jost , J. Kühnisch , F. Litzenburger , A. Lussi , and K. W. Neuhaus , “Clinical Validation of Near‐Infrared Light Transillumination for Early Proximal Caries Detection Using a Composite Reference Standard,” Journal of Dentistry 103s (2020): 100025. 34059307 10.1016/j.jjodo.2020.100025

[26] F. Mangano , A. Gandolfi , G. Luongo , and S. Logozzo , “Intraoral Scanners in Dentistry: A Review of the Current Literature,” BMC Oral Health 17, no. 1 (2017): 149. 29233132 10.1186/s12903-017-0442-x PMC5727697

[27] K. Doi , C. Yoshiga , R. Kobatake , M. Kawagoe , K. Wakamatsu , and K. Tsuga , “Use of an Intraoral Scanner to Evaluate Oral Health,” Journal of Oral Science 63, no. 3 (2021): 292–294. 34108300 10.2334/josnusd.21-0048

[28] K. C. Pentapati , P. Mishra , M. Damania , S. Narayanan , G. Sachdeva , and G. Bhalla , “Reliability of Intra‐Oral Camera Using Teledentistry in Screening of Oral Diseases—Pilot Study,” Saudi Dental Journal 29, no. 2 (2017): 74–77. 28490846 10.1016/j.sdentj.2017.03.002 PMC5411894

[29] K. Jung , K. Giese‐Kraft , M. A. Schlenz , B. Wöstmann , and C. Ganss , “Digital Plaque Monitoring: An Evaluation of Different Intraoral Scanners,” Journal of Dentistry 145 (2024): 104978. 38556195 10.1016/j.jdent.2024.104978

[30] M. Irving , R. Stewart , H. Spallek , and A. Blinkhorn , “Using Teledentistry in Clinical Practice as an Enabler to Improve Access to Clinical Care: A Qualitative Systematic Review,” Journal of Telemedicine and Telecare 24, no. 3 (2018): 129–146. 28092220 10.1177/1357633X16686776

[31] A. Queyroux , B. Saricassapian , D. Herzog , et al., “Accuracy of Teledentistry for Diagnosing Dental Pathology Using Direct Examination as a Gold Standard: Results of the Tel‐e‐Dent Study of Older Adults Living in Nursing Homes,” Journal of the American Medical Directors Association 18, no. 6 (2017): 528–532. 28236609 10.1016/j.jamda.2016.12.082

[32] L. Aquilanti , A. Santarelli , M. Mascitti , M. Procaccini , and G. Rappelli , “Dental Care Access and the Elderly: What Is the Role of Teledentistry? A Systematic Review,” International Journal of Environmental Research and Public Health 17, no. 23 (2020): 9053. 33291719 10.3390/ijerph17239053 PMC7729836

[33] A. Flores , S. A. Lazaro , C. G. Molina‐Bastos , et al., “Teledentistry in the Diagnosis of Oral Lesions: A Systematic Review of the Literature,” Journal of the American Medical Informatics Association 27, no. 7 (2020): 1166–1172. 32568392 10.1093/jamia/ocaa069 PMC7647318

[34] N. Rahman , S. Nathwani , and T. Kandiah , “Teledentistry From a Patient Perspective During the Coronavirus Pandemic,” British Dental Journal 229, no. 3 (2020): 1–4. 32801323 10.1038/s41415-020-1919-6 PMC7427495

